# Psychosocial Issues in Long-Term Survivors of Testicular Cancer

**DOI:** 10.3389/fendo.2019.00113

**Published:** 2019-02-25

**Authors:** Giuseppe Schepisi, Silvia De Padova, Delia De Lisi, Chiara Casadei, Elena Meggiolaro, Federica Ruffilli, Giovanni Rosti, Cristian Lolli, Giorgia Ravaglia, Vincenza Conteduca, Alberto Farolfi, Luigi Grassi, Ugo De Giorgi

**Affiliations:** ^1^Medical Oncology Department, Istituto Scientifico Romagnolo per lo Studio e la Cura dei Tumori IRCCS, Meldola, Italy; ^2^Psycho-Oncology Unit, Istituto Scientifico Romagnolo per lo Studio e la Cura dei Tumori IRCCS, Meldola, Italy; ^3^Medical Oncology Department, Santa Chiara Hospital, Trento, Italy; ^4^Unit of Biostatistics and Clinical Trials, Istituto Scientifico Romagnolo per lo Studio e la Cura dei Tumori IRCCS, Meldola, Italy; ^5^Hospital Psychiatry Unit, Department of Biomedical and Specialty Surgical Sciences, Integrated Department of Mental Health and Addictive Behavior, Institute of Psychiatry, St. Anna University Hospital and NHS Community Health Trusts, University of Ferrara, Ferrara, Italy

**Keywords:** testicular cancer, survivors, psychological concerns, sexual problems, reentry

## Abstract

Testicular cancer is the most frequent tumor in young males aged 15–39 years. As cure rates are currently around 90%, the prevalence of survivors is increasing. However, a disease-free condition does not necessarily correspond to a life free of physical and psychosocial health problems. The aim of this review was to explore psychosocial morbidity among testicular cancer survivors. A literature search was conducted in three electronic databases (PubMed, Medline, and Embase). The results of the search on cancer survivors were then combined with those of the search on psychosocial concerns and work performance. Eighty-four publications met the inclusion criteria. Physical, psychological, work-related problems and changing perspectives about work and life in general influenced life and career decisions among testicular cancer survivors. Individual health, sexual relationships and work problems, affect several important aspects of survival and significantly influence the QoL of long-term survivors.

## Introduction

Testicular cancer (TC), which represents 1% of male tumors, is the most frequent tumor in young males aged 15–39 years, and its incidence is increasing worldwide (1).

The most frequent histology is germ cell tumor (which represents 90–95% of cases): there are several subgroups of germ cell tumors: Seminoma (Pure, Spermatocytic), Embryonal Carcinoma, Choriocarcinoma, Yolk Sac Tumor, Teratoma (mature, immature, with malignant component). Some epidemiological risk factors has been detected: cryptorchidism, hypospadias, decreased spermatogenesis, familial history, or personal history of contralateral TC. Overall, this is often a treatable tumor, but prognosis and consequently mortality depend on the risk categories defined by the 1997 International Germ Cell Consensus Classification (Table 1) (2). TC can be cured by surgery, in case of localized disease, or by chemotherapy, in case of metastatic disease. Platinum-based chemotherapy regimens are the standard treatment because they allow to obtain complete responses, even in metastatic patients (3). As cure rates for TC are currently around 90%, the prevalence of TC survivors (TCSs) is increasing and their life expectancy is considered comparable to that of the age-matched male general population (4, 5). However, a disease-free condition does not necessarily correspond to a life free of physical and psychosocial health problems.

**Table 1:** Prognostic groups based on the IGCCCG Consensus Classification (2).

**Prognostic group**	**Seminoma**	**Non-seminoma**	**5 year survival**
Good	• Any primary site • Normal AFP • Any hCG and LDH • No non-pulmonary visceral metastases	• Testis/retroperitoneal primary • No non-pulmonary visceral metastases • AFP < 1,000 ng/mL • hCG < 5,000 IU/L (1,000 ng/mL) • LDH < 1.5 × ULN	~90%
Intermediate	• Any primary site • Non-pulmonary visceral metastases • Normal AFP • Any hCG and LDH	• Testis/retroperitoneal primary • No non-pulmonary visceral metastases • Tumor Markers S2 ◦ hCG 5,000–50,000 mIU/ml ◦ AFP 1,000–10,000 ng/ml ◦ LDH 1,5–10 × ULN	~75%
Poor	No patients	• Mediastinal primary + • Tumor Markers S3 ◦ hCG >50,000 mIU/ml ◦ AFP >10,000 ng/ml ◦ LDH >10 × ULN • Non-pulmonary visceral metastases	~45%

Although the majority of TCSs experience good levels of functioning and enjoy a health-related quality of life (HRQoL) comparable to that of the general population, a minority of survivors are faced with the long-term psychosocial effects and somatic sequelae of their disease history and previous treatments (platinum-based chemotherapy, radiotherapy and/or retroperitoneal lymphoadenectomy) (6–8).

It has been estimated that the overall incidence rate of late effects among TCSs is 66.3 per 1,000 persons/year, and that a higher risk is observed for hypercholesterolemia, infertility, and orchitis (9). Fertility issues, fatigue, chronic peripheral neuropathy, hearing loss, Raynaud-like phenomenon, tinnitus, cardiovascular toxicity, decreased pulmonary function, hypertension, and hyperthyroidism have also been reported (6, 10–19).

Furthermore, TCSs have a slightly higher risk than normal of developing germ cell tumors and/or treatment-induced non-germ cell tumors (15). There is also evidence that TC diagnosis and treatment can cause psychosocial problems in survivors (20) such as anxiety, fertility distress, fear of recurrence (21–23), all of which reduce overall life satisfaction and negatively affect social contacts and family relationships (20).

The aim of this review was to explore psychosocial morbidity among TCSs, focusing on 3 levels of concern: physical, psychological, and reentry, as conceptualized by Holland et al. (24).

## Methods

A literature search was conducted in three electronic databases (PubMed, Medline, and Embase) and original studies published up to October 2017 were identified. The term “survivor(s)” was combined with “testis cancer,” “testicular cancer,” and “germ-cell tumors” to facilitate the retrieval of abstracts about TCSs. The terms “physical,” “psychological,” and “quality of life” were used to search for material on physical and psychosocial issues. For work, the terms “work,” “job,” “worker(s),” “absenteeism,” and “reentry problems” were used.

In this narrative review, the results of the search on cancer survivors were then combined with those of the search on psychosocial concerns and work performance.

## Results

### Physical Issues

Physical concerns include continued preoccupation with illness and hypervigilance regarding minor symptoms, aches and pains, fears of disease recurrence or relapse, increased feelings of physical damage and infertility, and concerns about sexuality and attractiveness (24). Main physical concerns are summarized in Table 2. Over 70% of TCSs assessed their general health as good (8) and perceived their overall quality of life as equal to or slightly better than that of healthy men (6, 8, 29, 30). However, it has been seen that the quality of life of TCSs can be compromised by some disease-related health issues and physical limitations (30). According to some studies the greatest changes are physical (31, 32) and, among these, the main long-term sequelae are impairment of sexual life and fertility (29).

**Table 2:** Physical issues in long-term TCSs.

	**Physical issues**	**Percentage of patients**	**References**
Sexual problems	Problems with ejaculation	29–44	(25) (26)
		25.7	(27)
	Reduction of sexual activity	27.3	(27)
		9–24	(25) (26)
	Loss of desire	17.3	(27)
		7–20	(25) (26)
	Feeling less attractive	15	(28)

The uneasiness related to physical issues can take different forms. The experience of TC may compromise the person's sense of “invulnerability” and safety and replace it with fear of recurrence, concerns about other tumors, and death (33). Anxiety about follow-up diagnostic tests and cancer recurrence are known to be very common in cancer survivors and to persist a long time after the end of treatments (34, 35), despite the good prognosis for TC and the rarity of late recurrence (1–4% of cases) (36, 37). Fosså et al. (38) found that 17% of TCSs reported a worsening of anxiety related to relapse 2 years after the baseline measurement, whereas 36% of patients displayed improved global quality of life as compared with baseline Skaali et al. reported that almost one out of 3 TCSs reported fear of recurrence an average of 11 years after diagnosis (22). Moreover, higher levels of fear of recurrence (FoR) were significantly associated with higher levels of psychological distress, but not with cancer histotypes or treatment modalities. However, treatment-induced neurotoxicity, fatigue and severe somatic symptoms were significantly associated with level of FoR. The level of FoR was negatively correlated with quality of life (QoL) scores (22).

TC and related treatment strategies can cause both physiological changes and emotional reactions that affect or interfere with sexual functioning.

Impairment of sexual life and fertility represent the main long-term physical sequelae with respect to healthy controls (29), with sexual problems after TC therapy present in around 20% of patients (25, 39).

TC usually occurs during a man's most sexually active years when the impact of disease and treatments on sexual functioning, fertility, identity, and body image can be devastating (26). There is evidence that perceived attractiveness, retaining fertility, having children, and living with a partner are among the most important predictors of good health-related quality of life for men 3–13 years post-treatment (8). Dahl et al. reported that, although TCSs experience more problems with sexual drive, erection and ejaculation than healthy men, sexual satisfaction is not decreased and is even better than control for younger survivors (20–39 years). Moreover, whilst increasing age, lack of a partner, and high levels of anxiety are associated with compromised overall sexual function, this is also known to be true of males in general (26).

A varying percentage of TCSs report having physical sexual problems: orgasmic problems (10–20%); ejaculatory failure (29–44%), which is related to surgery in the retroperitoneal area; and erectile dysfunction (around 10%), which is linked to radiotherapy. In addition to physical sexual issues, some survivors also report psychosexual dysfunction after treatment such as decreased libido (7–20%), decreased sexual activity (9–24%) and dissatisfaction (5–20%) (40, 41). Some studies have reported different data on erectile dysfunction, the prevalence of which after TC is similar to that found in the general population (20, 26).

A review and meta-analysis of 36 studies sexual functioning after treatment for TC, covering a mean follow-up period of 2.0–6.9 years, revealed no deterioration over the course of time apart from a decrease in sexual desire and an increase in sexual satisfaction (40). Nazareth et al. (41) stated that, in general, sexual dysfunction linked to treatment of TC persists for about 2 years post-treatment, after which functioning seems to recover. In a recent Danish study, conducted in a cohort of 2,260 TCSs with a median follow-up of 17 years, a relationship among chemo—(bleomycin, etoposide, and cisplatin) radiotherapy and increased risk of erectile dysfunction was found (42).

Sexual dysfunction may be due to biological or psychological causes or a combination of both. According to Jonker-Pool et al. (40), a distinction can be made: impairment in physiologic domains such as erection and ejaculation are associated with extent of disease and treatment modalities (i.e., surgery, radiotherapy, or chemotherapy), while psychological domains such as sex drive and satisfaction are treatment-independent. However, treatment strategies for TC can result in physiologic changes and at the same time trigger emotional reactions. Thus, decreased sexual functioning (e.g., reduction in or inhibition of libido) may be due to treatment-related somatic factors such as fatigue, general malaise, hair loss, and excessive weight changes attributable to emotional factors including about sexual performance, fear of loss of control, and uncertainty about the future (12, 43).

Psychological factors arising from having a life-threatening, genitourinary disease play a strongly mediating (if not determining) role in sexual functioning; the traumatic experience of having cancer may affect the sexuality of TCSs, influencing more subjective aspects such as sexual desire, sexual activity, and sexual satisfaction (20, 40, 44).

Because of the symbolic nature of the testes, the loss of this organ may affect masculinity, sexual identity, and body image. Castration or (hemi)castration is linked to fantasies, beliefs, myths, and cultural values about the testes that can have a severely traumatic effect and psychological consequences on the person/patient. Thus, concerns related to sexual and reproductive functioning may generate feelings of inadequacy, hopelessness, and emotional distress (45).

After removal of a testicle by orchiectomy, TCSs may have long-lasting feelings of loss or shame. Skoogh et al. (46) found that such feelings were more common among younger and single men than among older and non-single men. There was no correlation between feelings of loss or uneasiness and shame and having or not having a prosthesis, although offering a testicular prosthesis may help to reduce the trauma induced by this experience.

TC involves a male organ that is highly associated with perceptions of masculinity, attractiveness and body image. Patients undergoing removal of a testicle may see it as a disembodying procedure, especially during the period in their life when there is a heightened fixation on the “perfect body” and a striving for physical fitness (45).

Body image is a seldom explored topic in TC survivorship. In a work by Rossen et al., negative changes in body image, i.e., perceived reduced masculinity, following TC and its treatment, were reported in 17% of long-term TCSs and were associated with various aspects of sexual dysfunction, i.e., reduced sexual interest, reduced sexual activity, reduced sexual enjoyment, erectile dysfunction, ejaculation dysfunction, and increased sexual discomfort (28). Similar results have been reported in other studies. Tuinman et al. (47) found that 16% of survivors expressed concern about their body image and reported feeling anxious at the thought that other people notice the missing testicle. Rudberg et al. (48) reported that 15% of Swedish TCS felt less attractive. Thus, although the surgical removal of a testicle generally has a negative impact on body image, this tends to less over time (21) and most TCSs do not report feeling less attractive (8) or less masculine than before their experience of TC (38).

Another aspect that affects the emotional experience of TC patients is the meaning they give to the disease. TC attacks an organ intrinsically associated with sexuality and reproduction at a time of life when sexual desire and performance, sense of masculinity, body image, and fertility are central issues (49). A Norwegian study conducted on a cancer survivor population (including TCSs) reported a significant reduction in paternity in the TCSs compared to non-cancer males (27).

Recently, a Greek study of 53 TC patients submitted to full bilateral, non-nerve-sparing post-chemotherapy retroperitoneal lymph node dissection (RPLND) observed that orgasmic function, intercourse and overall sexual satisfaction were significantly impaired post-operatively (50). However, as a subjective perception, a substantial number of patients reported higher levels of sexual desire and no difference in erectile function poorer orgasmic function and satisfaction post-operatively (50).

In a Serbian cross-sectional study involving 202 TCSs, 27.3% experienced decreased sexual function compared to the period before chemotherapy, 20.8% reported no erectile function impairment and 25.7% had problems with ejaculation. Loss of desire was reported by 17.3% of TCSs (51).

An Italian study evaluating the effects of several kinds of treatment on erectile function found that only adjuvant radiotherapy was as an independent predictor of non-recovery of normal function. Adjuvant chemotherapy alone, chemotherapy plus RPLND or RPLND alone did not significantly impair the recovery of normal erections (52).

### Psychological Concerns

Although there is evidence to suggest that the majority of cancer survivors adjust well in terms of QoL and psychological well-being, emotional problems have been reported in a substantial minority of long-term TCSs (53). Main psychological concerns are summarized in Table 3. The experience of cancer and long-term physical sequelae of treatment can affect the psychological well-being and may lead to increased levels of psychological distress in those living with a history of cancer (57, 58). Psychosocial morbidity among cancer survivors include an increased sense of vulnerability and uncertainty about the future, feelings of personal inadequacy, fear of social rejection and stigmatization, anxiety, depression, and symptoms of post-traumatic stress disorder (24). Increased levels of anxiety and depression may also be present years after diagnosis (59, 60). A recent study by Inhestern et al. (61) confirmed the conclusions of a meta-analysis in long-term cancer survivors conducted by Mitchell et al. in which anxiety, rather than depression, was a more widely perceived problem in long-term cancer survivors than in healthy controls (62).

**Table 3:** Psychological issues in long-term TCSs.

**Psychological issues**	**Percentage of patients**	**References**
Fear of recurrence	33	(20)
	17	(36)
Increase in anxiety	25	(54)
	19	(55)
	6.1	(56)
Depression after treatment	20	(55)
	9–11	(54)
	7.9	(56)

Several studies on the lives of TC survivors have found that the psychosocial aspects of the disease, such as anxiety about the future, coping and work reentry, are more important determinants of distress, morbidity and QoL than the type of treatment undergone and the time since its completion. It is thus possible that subjective evaluations are more important determinants of functioning and contributors to distress than a patient's actual medical history (7, 55, 63, 64).

There is evidence that cancer may precipitate post-traumatic stress (PTS) conditions, including PTS symptoms or PTS disorders (PTSD). Literature also show that cancer may also facilitate post-traumatic growth such as positive perceptions of oneself, emotional growth, improving relationships with others, and greater appreciation of life (65–67), although little is known about this area is TC.

Several studies have shown that TC patients treated with chemotherapy are at risk of long-term lower cognitive performance (22, 68–70). Chemotherapy, especially platinum-based treatment, is associated with paraesthesia, hypogonadism, hypercholesterolemia, and hypertension (71), and also with memory problems and lower cognitive performance in TCSs (68). This condition, known as “chemo-brain” or “chemo-fog” has been described in other tumor types (72). In some cases, the prevalence of cognitive difficulties in TCSs is unexpectedly high, especially in terms of neuropsychological outcomes (73). Amidi et al. (74) found impairments related to verbal learning and memory (29–33% of TCSs), visual learning and memory (14–28%), processing speed (8–24%), executive functioning (17%), and attention and working memory (4–15%). However, it is worthy of note that no correlation between cognitive impairments and type of treatment has been identified (22, 68–70, 74, 75). Fung et al. consider that cognitive impairment could be related to anxiety and depression that are prevalent in this kind of patients (76).

Some studies evaluated the influence of different treatment modalities on quality of life: men who received the most aggressive treatment perceived the lowest HRQoL (8, 77). Each individual's experience with cancer is different. Cancer type and stage, type and severity of treatment, and subsequent physical effects may all contribute to how survivors “live” the experience of cancer, as well as to their levels of distress, personal growth, and QoL (78). TC, albeit curable in a high percentage of cases, has some aspects that make it an invasive emotional event and a particularly distressing experience. It mainly affects young men aged between 15 and 45 years during a central phase of their life cycle in which they are still constructing their own personal identity. The threat to existential continuity represented by cancer and the profound effect it has on body image and on personal values make this evolutionary transition a difficult one.

This in an important period of life, characterized by major life changes and specific developmental tasks. Indeed, these men are at or are near their prime of life, when interpersonal relationships, long-term work goals, and the desire to start a family may be major concerns (21, 54, 79, 80). Moreover, in this period of life health is generally taken for granted and life-threatening illnesses and dying are rarely considered possibilities (33).

The experience of TC may continue to influence the well-being of survivors and to interfere with the normal course of daily life months or even years after they are cured (54).

Physical and psychological consequences of treatment may interfere with life plans made before cancer, obliging survivors to review their short- and long-term goals. This may lead to impaired psychosocial functioning and more cancer-related distress (33).

A review of the literature on psychological and social domains showed that the majority of TCSs experience good levels of functioning and good post-therapeutic QoL, although a small percentage reported psychosocial problems such as anxiety, depression, fertility distress, sexual problems, and work-related problems (21, 40).

Qualitatively strong studies reviewed by Fleer et al. (21) indicated that the levels of psychological distress reported by TCSs varied between 9 and 27% (8, 23, 81). The study by Dahl et al. found that around 25% of TCSs became more anxious after diagnosis and treatment and that the distress experienced by TCSs is significantly higher than that of controls (81). However, other studies had different outcomes, e.g., a survey on QoL reported a slightly poorer mental health in TCSs than in a control group (7).

In the past, numerous studies have reported that the most frequent symptoms of emotional distress are tension, anxiety, restlessness, nervousness, and health worries (79). It has been seen that long-term TCSs continue to have significantly higher levels of anxiety (but not depression) many years after treatment compared to age-adjusted healthy males (1, 12, 23, 56). Substantially increased levels of anxiety among TCSs with respect to controls are associated with peripheral neuropathy, fear of recurrence, economic concerns, alcohol abuse, sexual difficulties, younger age at diagnosis, and a history of treatment for mental problems (12).

Among the possible causes of the increased anxiety experienced by a considerable proportion of TCS are a feeling of unsafety (54), a paradoxical perceived loss of protection by medical providers, a decrease in medical surveillance, and the perception of being completely on one's own. Symptoms of anxiety often occur before follow-up visits. Although the prevalence of depression among long-term TCSs corresponds to that observed in the general population (6, 56), some studies report a different frequency of self-reported depressive symptoms (79, 82). For example, Dahl et al. reported that depression was prevalent in 9–11% of TCSs up to 5 years after the end of treatment (56).

Thus, the overall picture regarding depression in TCSs is somewhat unclear (82). In general, differences in study results can be attributed to different aspects of distress evaluated, sampling differences in survivors, and the use of different validated questionnaires. A recent study assessed the prevalence of anxiety/depression in long-term TCSs, reporting anxiety in 6.1% of survivors and depression in 7.9%. Younger age at diagnosis and a shorter time since diagnosis were significantly associated with higher anxiety (83). A Polish study evaluated levels of anxiety/depression in 111 TC patients (57 undergoing chemotherapy and 54 patients at least 6 months after treatment). The prevalence of anxiety disorder was 40% during chemotherapy and 18.5% after treatment. Depression was present in 14.6% of patients during chemotherapy and in 9.3% after treatment. The prevalence of aggressiveness was 5.6% in patients during chemotherapy and 18.9% in the post-treatment group (84).

An Australian study conducted on 486 eligible TC survivors found small but significant increases in mean levels of anxiety and depression, a greater prevalence of extremely severe anxiety (19%) and depression (20%), and significant deficits in mainly mental aspects of generic HRQoL. The majority of TCSs reported one or more unmet needs regarding existential issues, more frequent than in breast and gynecological cancer survivors and probably correlated to the young age of the TCSs (64).

A diagnosis of cancer and associated treatments may represent a potentially ongoing threat and trigger recurring challenges. Indeed, this life-threatening illness is conceptualized as a potentially multidimensional traumatic event that risks compromising body integrity, leading to disability, disfigurement, pain and loss of social and occupational functioning, and creating dependence on others (24). Although most cancer survivors do not meet the criteria to be diagnosed with post-traumatic stress disorder, they may nevertheless report painful re-experiencing of the cancer diagnosis and treatment-related events. Traumatic stress symptomatology in terms of intrusive thoughts about the disease, avoidance of reminders of cancer, and hyper-vigilance are commonly reported by survivors after completion of treatment (85).

Little is known about cancer-related stress symptoms in TCSs. Some studies (31, 55) have focused on the cancer-related stress symptoms of intrusion and avoidance, the core symptoms of PTSD (86). Fleer et al. (55) reported that a minority (13%) of TCSs experience clinically elevated levels of cancer-related stress symptoms. In particular, TCSs with a lower level of education and unemployed survivors reported higher levels of cancer-related stress symptoms than their counterparts. The authors also reported that the impact of the illness felt by TCSs on their current lives and their anxiety about the future contributed more significantly to distress than objective illness variables. Mykletun et al. (31) observed that TCSs who experienced more TC-related stress were more likely to report reduced QoL, but concluded that the stress was not attributable to treatment strategies. A recent study reported that in a sample of TCSs who were 11 years post-diagnosis, just over 10% had either subclinical or full PTSD. Probable PTSD was not related to time since TC diagnosis, but was significantly associated with cisplatin-related side effects, probable anxiety disorder, and poor self-rated health at 11 years post-diagnosis (87).

A recent study by Norwegian researchers evaluated the prevalence of chronic fatigue among 812 TC survivors. The risk of this disorder increased 3- to 4-fold for high levels of neuropathy vs. no neuropathy, and 2- to 3-fold for high levels of Raynaud-like phenomena and when testosterone levels were in the lowest quartile. Conversely, moderate to high physical activity had a protective effect against the syndrome (88).

Neuroticism in TCSs undergoing long-term follow-up is significantly associated with somatic and mental morbidities, self-esteem, concerns about not being able to father children, sexual problems, use of alcohol, sedatives and hypnotics, frequent visits to their G.P., and seeing a psychologist/psychiatrist (63).

### Partnered Relationships

Cancer has considerable psychosocial implications related to the impact of the disease and its treatment on the individual from a psychological and spiritual point of view, and also from the perspective of interpersonal and social relationships (89, 90). The disease, far from being individual experience, also exerts a profound effect on patients' families. In particular, the partners of cancer patients are subject to a wide range of both emotional and practical repercussions throughout the course of the disease. For couples who face the survivorship phase, the main tasks include resuming a sexual relationship, discussing changes in life plans, deciding on health behavior changes, dealing with disease and treatment-related late effects that may influence patient functioning, managing worry about disease recurrence, and reflecting on the impact the cancer has had on themselves and the relationship (91).

Affective-relational life is an important theme in TC survivorship. It has been found that romantic relationships are associated with both positive (e.g., improved physical and emotional function) and negative aspects (e.g., new conflicts) (91). The majority of long-term TCSs and their wives report that their experience with cancer draws them closer as a couple, strengthening their mutual ties, trust, understanding, commitment to each other, and intimacy (92, 93). This aspect was also highlighted in a review on sexual functioning of TCSs and their partners (94). Although sexuality may be restricted or impaired by the experience of cancer, the decline in sexual satisfaction is usually very limited (44). As Jankowska explained, it is possible that patients who are facing a life-threatening disease may reorient their life's priorities and values with regard to sexuality and the relationship with their partner, reaping positive benefits such as greater intimacy and closeness (94). Thus, the psychological and affective aspects of the dyadic dimension may play a protective role in sexual function.

Relationship status (partnered vs. unpartnered) can play an important role in adjustment outcomes (91). Men who were involved in a relationship at the time of TC describe a better physical and emotional adaptation to the cancer experience (95, 96). Tuinman et al. also described positive outcomes and a higher level of functioning for survivors with a continuing relationship after diagnosis. In particular, they reported greater levels of social support, self-esteem and overall mental health compared with single TCSs and survivors who met their partner after the completion of treatment (97).

There is ample evidence in the literature about the relational difficulties experienced by individuals with a history of TC. For young adults involved or thinking about becoming involved in intimate relationships, the effect of TC treatment on sexual function, fertility, and overall future health may represent significant barriers to successful romantic and sexual relationships. Carpentier et al. describe 4 recurring themes related to testicular cancer diagnosis that can interfere and influence satisfaction in the romantic relationships of survivors: feeling different, viewing their differences as “damaged goods,” struggles with cancer-related disclosures, and feelings of embarrassment (98).

Survivors unpartnered during treatment express worry about their history of cancer affecting future interpersonal relationships (95, 99).

New relationship difficulties reported by TCSs and spouses concern communication problems centered on a fear of talking about the cancer, problems in understanding and expressing feelings with their respective partners, the possibility of recurrence, and implications for the future (93).

In some cases, TC exacerbates pre-existing relationship conflicts or creates new conflicts which ultimately lead to relationship dissolution. A cancer experience leads to a greater appreciation of life in which conflicts no longer have a place, thus leading survivors to end conflict-plagued relationships (91). The majority of TCSs (70–90%) are in partnered relationships when TC is diagnosed and the majority of follow-up studies show that the rate of divorce and broken relationships for TCSs is 5 to 10% (100). Conversely, Joly et al. reported that friendships were more likely to remain intact for TCSs than for controls (29). Similar results were obtained by Syse in terms of marriage percentages (101).

### Reentry Problems

Social and functional life (work and study) issues faced by cancer survivors may include difficulties in transitioning from patient to healthy status, being regarded by others as “special,” feeling that one's job is not secure, experiencing discrimination and/or negative peer and employer attitudes (24). Several studies have been conducted to investigate reentry problems in cancer survivors and employment rate data vary considerably, ranging from 35 (102) to 67% for long-term survivors (103, 104). The differences in percentages may be attributable to different cancer types examined. However, a Finnish study reported only a 9% lower employment rate than that of the cancer-free population (105). This discrepancy may be correlated with the fact that people with a higher level of education have a greater chance of being employed after their cancer diagnosis than less educated patients job type is also a factor, e.g., manual labor is negatively associated with a return to work due to its physically strenuous nature (106, 107). With regard to TCSs, coping behavior is not only needed for human relationships, but also for work situations. Rutskij et al. reported that TCSs with poorer avoidance coping skills fared worse in terms of paired relationships and paid work than TCSs with a better approach to coping (108). Another study showed that TCSs diagnosed <5 years earlier reported more absenteeism than controls, whereas there was no difference between controls and survivors diagnosed >5 years earlier (109). An interesting study conducted among breast, testicular and prostate cancer survivors in Northern Europe (NOCWO trial) did not reveal any differences in work engagement between cancer survivors and other employees, despite all of the problems reported by survivors, i.e., poorer health status, physical QoL, and work ability, more anxiety, and significantly higher neuroticism (110).

Within this context, recent studies have highlighted the importance of post-treatment psychosocial and behavioral interventions (111). In particular, knowledge that one's job is secure should be acknowledged as a prerequisite for normal living conditions (112). Improving communication at the workplace and developing supportive leadership practices are needed to avoid isolating behavior in cancer survivors (113).

## Conclusions

TC is perhaps the paradigm of cancers with problems related to a long-term survival. There are 2 fundamental reasons for this, i.e., the curability of a high number of patients, leading to better long-term survival, but also the onset at a young age, leading to problems that differ from those arising from tumors diagnosed later in life. Such problems, including individual health, sexual relationships and work problems, affect several important aspects of survival and significantly influence the QoL of long-term survivors. Recently, a web-based computer-tailored intervention, the Kanker Nazorg Wijzer (Cancer Aftercare Guide), was developed in the Netherlands with the aim of providing psychosocial and lifestyle support for cancer survivors. It not only provides the most appropriate advice regarding physical activity, diet etc., but also measures psychosocial well-being by assessing QoL, psychological distress, mental adjustment to cancer, fatigue, work limitations, and social support. This tool is not yet suitable for use for TCS-related problems (114, 115).

As far as we know, QoL evaluations in long-term TCSs have only been conducted in single country trials. Recently, however, some studies have begun assessing the feasibility of collecting QoL data among TCSs recruited from different countries (116, 117). To better understand the impact of TC on QoL it is important to know how sociocultural differences in sexuality, masculinity and fertility influence the survivors. This problem exist and is still very wide. Educational events, patients associations and the development of Cancer Aftercare Guides, as like as the abovementioned Dutch project, could provide some solutions to a better awareness about the importance of QoL in TCSs.

## Author Contributions

GS and SD have collaborated in the conception, in the data retrieval, and in the drafting of the text. DD and CC have collaborated in the revision of the text and in the completion of the bibliographic research. EM, FR, GRo, CL, GRa, VC, AF, LG, and UD revised the manuscript.

### Conflict of Interest Statement

The authors declare that the research was conducted in the absence of any commercial or financial relationships that could be construed as a potential conflict of interest.
